# Lectures on Dietetics and Dyspepsia

**Published:** 1886-12

**Authors:** 


2. LECTURES ON DIETETICS AND DYSPEPSIA. *By* SIR WILLIAM ROBERTS, M.D., F.R.S. *Second Edition. Small 8vo, pp. ix, 92. London:* SMITH, ELDER & COMPANY. *New York:* G. P. PUTNAM'S SONS. *Chicago:* A. C.McCLURG & COMPANY. *1886.*

1. This little manual follows the plan, adopted in so many of our recent works, of giving the differential diagnostic symptoms in parallel columns. Thus, under the head of diseases of the mouth and throat, we find œdema glottidis differentiated from croup, thoracic aneurism, asthma and retro-pharyngeal abscess. Again, under acute general disease, we find measles compared with variola, scarlet fever, roseola, and typhus.

The teacher will find this little work useful, as by it he may appeal to the eye as well as the ear of the student.

A few minor errors are to be observed, such as "typhus fever," but in the main the work is reliable, having been compiled from the best sources.

2. This little work is fragmentary. The first lecture deals with dietetics in general, and shows extensive reading and careful thought. The fifth lecture is a valuable monograph on the nature, causes and treatment of acid dyspepsia. The three remaining lectures embody the author's views and experiments on the accessory articles of food, tea, coffee, alcohol, etc., and their relation to digestion. His views are in many respects different from those commonly accepted.

They are, however, theoretically correct, and will doubtless stand the test of practical application. The work is a valuable addition to the literature of dietetics.

The addition of the terms "collagenous," "oligopepsia," and "horrification" to our nomenclature may be regarded as of doubtful utility.

